# The HEM Lines: A New Library of Homozygous *Arabidopsis thaliana* EMS Mutants and its Potential to Detect Meiotic Phenotypes

**DOI:** 10.3389/fpls.2018.01339

**Published:** 2018-09-19

**Authors:** Laia Capilla-Perez, Victor Solier, Virginie Portemer, Aurelie Chambon, Aurelie Hurel, Alexia Guillebaux, Daniel Vezon, Laurence Cromer, Mathilde Grelon, Raphael Mercier

**Affiliations:** CNRS, Institut Jean-Pierre Bourgin, INRA, AgroParisTech, Université Paris-Saclay, Versailles, France

**Keywords:** forward screens, mutagenesis, meiosis, EMS, mutant collection, *Arabidopsis*

## Abstract

Genetic screens have been crucial for deciphering many important biological processes, including meiosis. In *Arabidopsis thaliana*, previous forward screens have likely identified almost all the meiotic genes that when mutated lead to a pronounced decrease in fertility. However, the increasing number of genes identified in reverse genetics studies that play crucial roles in meiosis, but do not exhibit strong phenotypes when mutated, suggests that there are still many genes with meiotic function waiting to be discovered. In this study, we produced 897 *A. thaliana* homozygous mutant lines using Ethyl Methyl Sulfonate (EMS) mutagenesis followed by either single seed descent or haploid doubling. Whole genome sequencing of a subset of lines showed an average of 696 homozygous mutations per line, 195 of which (28%) modify a protein sequence. To test the power of this library, we carried out a forward screen looking for meiotic defects by observing chromosomes at metaphase I of male meiosis. Among the 649 lines analyzed, we identified 43 lines with meiotic defects. Of these, 21 lines had an obvious candidate causal mutation, namely a STOP or splicing site mutation in a gene previously shown to play a role in meiosis (*ATM, MLH3, MLH1, MER3, HEI10, FLIP, ASY4, FLIP, PRD2, REC8, FANCL*, and *PSS1*). Interestingly, this was the first time that six of these genes were identified in a forward screen in *Arabidopsis* (*MLH3, MLH1, SGO1, PSS1*, *FANCL*, and *ASY4*). These results illustrate the potential of this mutant population for screening for any qualitative or quantitative phenotype. Thus, this new mutant library is a powerful tool for functional genomics in *A. thaliana*. The HEM (Homozygote EMS Mutants) lines are available at the Versailles *Arabidopsis* stock center.

## Introduction

Genetic screens have improved our knowledge of the genetic basis of biological processes. However, to identify all the genes involved in a biological pathway screening strategies must be sensitive, specific and feasible. In this context, the two classic approaches, which are still widely used, are forward and reverse genetics. On one hand, forward genetics can be used to decipher specific pathways without *a priori* knowledge of the genes involved. This is achieved by screening the phenotype of a population with random mutations throughout the genome, which alter gene function and thus give an identifiable phenotype. On the other hand, the principle of reverse genetics is to test the function of a specific candidate gene by analysing the consequences of its disruption on the process of interest.

Different types of chemical, biological and physical mutagens have been used to generate mutant collections for forward and reverse genetics (reviewed in Page and Grossniklaus, 2002; Nawaz and Shu, 2014). Ethyl Methyl Sulfonate (EMS) is the most common mutagen as it is very easy to use and can produce very high mutagenesis rates compared with other methods (reviewed in Sikora et al., 2011). EMS has an alkylating effect that mainly induces point mutations with G/C to T/A transitions, as seen for example in rice (Till et al., 2007), maize (Till et al., 2004), and *Arabidopsis* (McCallum et al., 2000; Martín et al., 2009). Point mutations have the potential to not only produce loss-of-function mutants but also weak or separation-of-function alleles (e.g., Séguéla-Arnaud et al., 2015). Thus analysis of these mutants can be used to functionally characterize essential genes.

Meiosis is a specialized cell division where two rounds of chromosome segregation follow one round of DNA replication leading to the production of haploid cells that are essential for sexual reproduction. The list of genes described to be involved in meiosis has steadily increased as a result of various genetic screens (see Mercier et al., 2015 and references therein, 2015). *Arabidopsis* first emerged as a model in the late 90s when T-DNA insertion lines with meiotic defects were first characterized (Peirson et al., 1997). Subsequently, large-scale forward screens in *Arabidopsis* (e.g., De Muyt et al., 2009) identified meiotic mutants by looking for mutant lines with reduced fertility as a result of meiotic defects. This strategy led to the description of a number of genes involved in meiosis, however, it was also biased toward genes whose disruption produces very pronounced meiotic defects. More recently, meiotic genes were identified in suppressor screens, for example by observing fertility restoration in *zmm* mutants (Crismani et al., 2012; Girard et al., 2014; Séguéla-Arnaud et al., 2015; Fernandes et al., 2018). The phenotypes of these mutants consist of an increase in crossover number, which is not easily observable at a macroscopic level, preventing identification in forward screens.

In parallel, an increasing number of important meiotic genes have been identified from reverse genetics screens such as *MLH1* (Dion et al., 2007), *MLH3* (Jackson et al., 2006), *SGO1* (Cromer et al., 2013; Zamariola et al., 2013), *PSS1* (Duroc et al., 2014), *PCH2* (Lambing et al., 2015), and *RPA1* (Osman et al., 2009), among others. These mutants are characterized by subtle fertility defects and have not yet been identified in any forward screens. This therefore suggests that a number of meiotic genes, whose inactivation leads to subtle meiotic defects, could have been missed in previous forward screens based on reduced fertility observable at a macroscopic level.

Here, we produced a total of 897 homozygous *Arabidopsis thaliana* EMS mutant lines with >170,000 mutations leading to changes in protein sequences and identified meiotic defects in 43 lines. These results demonstrate the usefulness of these HEM (Homozygote EMS Mutant) lines that can be used to detect either qualitative or quantitative phenotypes. Thus this new mutant collection is a very useful resource for functional genomics and applied research in *A. thaliana.*

## Results

### Generation of the HEM Lines: Two Collections of Homozygous *Arabidopsis thaliana* EMS Mutants

**FIGURE 1:**
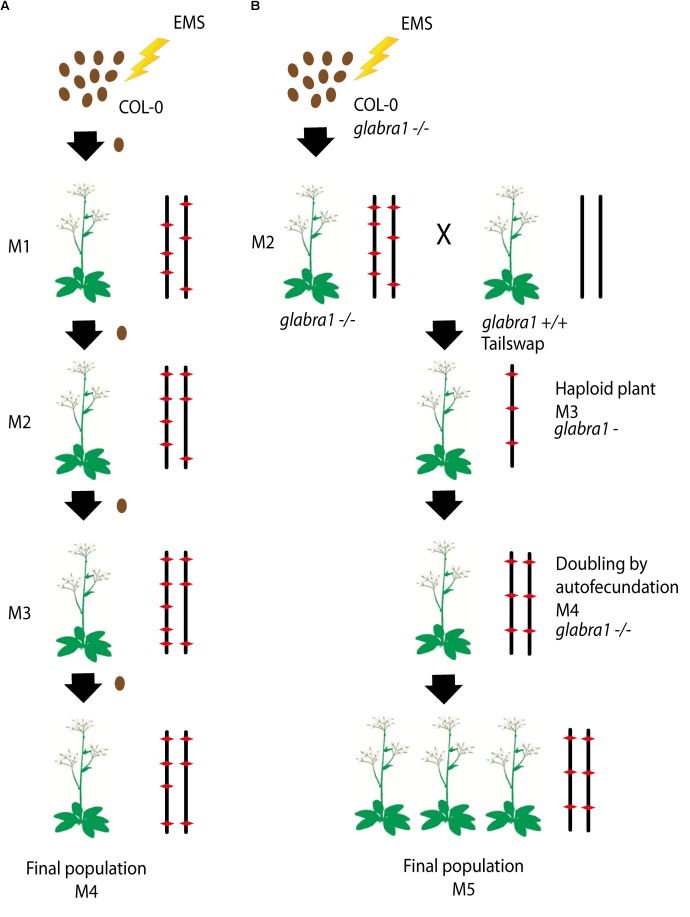
**(A,B)** Schematic diagram of the approaches used to generate each subset of the homozygous EMS mutant lines: (i) Single seed descent (SSD) **(A)** and (ii) doubled haploids (DH) **(B)**. **(A)** For the SSD population, a set of Col-0 seeds were mutagenized by EMS and four different generations were grown from them by selecting a single seed in each generation. **(B)** The DH lines were obtained by mutagenizing a set of Col-0 GALBRA-1 seeds. M2 were then crossed to the Tailswap strain and the haploids of the next generation were selected by the absence of trichomes produced by the GLABRA- 1 mutation. Diploids were then obtained by spontaneous doubling from self-fertilization of the M3 plants. Chromosomes representing the genetic constitution (EMS mutations represented by a red diamond) are displayed for each generation.

To produce the HEM lines we generated two collections of almost fully homozygous lines using two different strategies: (i) single seed descent (SSD) and (ii) doubled haploids (DH) (**Figures 1A,B**).

For the SSD subset we applied EMS to wild type Col-0 seeds and produced four successive generations by self-fertilization using a single seed in each generation (SSD) (**Figure 1A**). Six parallel rounds of mutagenesis followed by SSD were carried out, giving a total of 698 independent mutant lines (**Table 1**). With this approach, we expected to obtain a level of homozygosity of 87% in the 4th generation (M4) that was screened (see below). The M5 seeds are available at the Versailles *Arabidopsis* stock center.

**Table 1:** The different series of independent mutagenesis carried out to generate each of the HEM subsets.

	SSD collection			DH collection		
Series	Lines in the collection	Lines screened	Meiotic mutants	Lines in the collection	Lines screened	Meiotic mutants
1	60	58	0	199	199	18
2	90	55	0			
3	47	43	0			
6	140	–	–			
10	302	261	25			
11	59	26	0			

*The number of mutant lines produced for each subset of the HEM lines (SSD and DH) is shown with the different independent mutagenesis series produced, the number of lines screened for subtle meiotic phenotypes at metaphase I and the number of meiotic mutants found in each subset.*

For the second population, the strategy was to generate homozygous mutagenized lines by haploidization followed by genome doubling (Doubled haploid, DH) (**Figure 1B**). We applied EMS to Col-0 seeds with a homozygous mutation in the *GALBRA-1* gene (*GL1)*, which is characterized by the absence of trichomes (Marks and Feldmann, 1989). The first generation plants (M1) were selfed and the next generation (M2) was crossed to a haploid inducing strain (the CENH3 Tailswap line) to obtain haploid descendants (Ravi and Chan, 2010). M3 haploid plants were visually identified due to the absence of trichomes conferred by the *gl1* mutation (Portemer et al., 2015) and reproduced by self-fertilization, which spontaneously produced diploid seeds (M4). Using this approach, we expected to obtain virtually completely homozygous mutant lines. Finally, M4 seeds were propagated to obtain a total of 199 M5 independent lines (**Figure 1B**).

### Analysis of Mutation Frequencies in the HEM Lines

To estimate the number of mutations in the HEM populations, we sequenced 47 mutant lines using Illumina: 25 SSD lines (series 10) and 22 DH lines. Among these, 41 lines showed a meiotic defect (see section below) and 6 lines without meiotic defects were randomly chosen among the DH lines.

**FIGURE 2:**
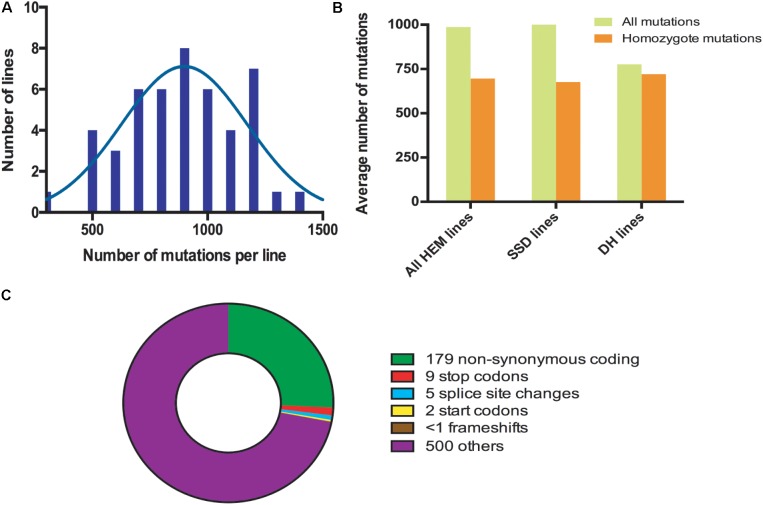
**(A)** Distribution frequencies of the total number of mutations per HEM line. **(B)** The average total number of and homozygous mutations per line, comparing the HEM lines and each of the subsets (SSDs and DHs). **(C)** Average number of each type of mutation predicted to change the sequence and/or knockout a protein in one HEM line. “Others” category includes: synonymous coding, intergenic, intragenic, intron, and synonymous stop mutations.

The HEM populations showed a total mean number of 897 mutations per line, ranging from 500 to 1,500, with a normal distribution (**Figure 2A**). Of these 897 mutations, 99% were G > A or C > T transitions. When this value was compared for each of the subsets obtained, the SSD lines had more mutations (1,003 mutations per line) than the DHs (with 776 mutations per line; *T*-test, *p* < 0.05^∗^; **Figure 2B** and **Table 2**).

**Table 2:** Number of mutations detected in the HEM lines and in the SSD and DH subsets.

	HEM (*n* = 47)	SSD (*n* = 25)	DH (*n* = 22)
All Mutations	897	1,003	776
Homozygous mutations	696 (81%)	676 (70%)	720 (94%)
Homozygous mutations that change protein sequences	195	193	199
Homozygous non synonymous coding mutations	179	177	182
Homozygous mutations that may affect protein function^∗^	14	15	14

*^∗^This category includes premature stops, splice site changes, and frameshifts.*

The percentage of fixed mutations, was different for each of the subsets due to the different approaches used (SSD and DH; **Figure 2B** and **Table 2**): in the SSD lines 70% of the mutations were fixed (676 mutations per line on average), which was lower than the 87% expected for those lines, perhaps due to counter-selection. In the case of the DH lines, we obtained an average of 94% homozygous mutations (720 mutations per line on average). The six randomly chosen lines had a similar number of mutations than the lines having a meiotic phenotype (690 vs. 697 mutations per line, respectively).

Considering that the DH lines were produced by doubling the genome of haploid plants, we expected complete fixation of the mutations and this is what we observed in 16 of the 22 lines analyzed (>95% of detected mutation). However, in six lines the percentage of homozygous mutations was 80% (on average) suggesting that the haploidization was not successful or that cross-pollination occurred during their production. Regardless, these lines still show a very high level of homozygosity, equivalent to that in the SSD lines.

In all the HEM lines (SSD and DH), 28% of the fixed mutations (an average of 195 mutations per line) modified a protein sequence (**Figure 2C** and **Table 2**). This category includes: non-synonymous coding mutations that change a single AA (179 mutations per line, on average); small indels leading to frame shifts (<1 per line, on average); new stop codons (nine per line, on average); new start codons (two per line, on average) and splice site changes (five per line, on average). Considering only the mutations resulting in frameshifts, new stop codons or splice site changes, 14 mutations per line should severely disrupt gene functions (**Figure 2C** and **Table 2**).

In summary, when a single line of the HEM library is screened, the effect of 195 homozygous mutations causing an amino acid change can be examined, at least 14 of which are predicted to knock out the function of the protein.

### A Screen for Subtle Meiotic Defects

The HEM populations were then tested in a forward genetic screen targeting subtle meiotic phenotypes with the aim of identifying new genes involved in the meiotic process. For this we used two different approaches: (i) Alexander staining to detect dead pollen grains (Alexander, 1969) and then observations of meiotic chromosomal behavior (using chromosome spreads) in the selected lines, or (ii) direct observation of meiotic chromosomal spreads without a pre-screen.

All of the 199 M5 mutant lines from the DH subset were first pre-screened by Alexander staining. The meiotic chromosomal behavior was then examined in lines with more than 10% dead pollen grains. In the case of the SSD lines, we screened 539 M4 mutants (77% of the total 698 lines produced) by directly observing meiotic chromosomal behavior at metaphase I. Of these, the lines with a minimum of 20 cells at metaphase I captured were considered as screened, which resulted in a total of 450 mutant lines. For each of the mutants with a meiotic defect at metaphase I, we verified that the same phenotype was observed in the next generation. To optimize the screening procedure, we focused on metaphase I: (i) cells at that stage are relatively easy to find and (ii) most meiotic defects can be detected at metaphase I (e.g., modifications in crossover number or distribution, chromosome alignment defects and DSB repair defects). A drawback is that some defects cannot be observed at that stage (e.g., premature loss of cohesion, meiosis II spindle defects, cell cycle defects) and would be missed in this screen. However, we occasionally detected meiosis II defects that were included in the study.

We identified a total of 43 lines with various meiotic defects (18 DH lines and 25 SSD lines), representing 9% (18/199) of the screened DH lines and 6% (25/450) of the SSD lines. However, we observed an important difference among the six different series of mutagenesis used to produce the SSD subset (**Table 1**): 10% of the lines (25/261; **Table 1**) had meiotic defects in series 10, whereas no meiotic defects were observed in the other series produced (1–3, 6 and 11; 0/189). This variability could reflect differences in the efficiency of the mutagenesis due to slight variations in experimental conditions (e.g., room temperature, age of the seeds…) that may influence the final outcome of EMS mutagenesis. Therefore, the high number of meiotic mutants observed in the SSD series 10 and the HD lines suggests that these series are especially suitable for carrying out other forward genetic screens.

**FIGURE 3:**
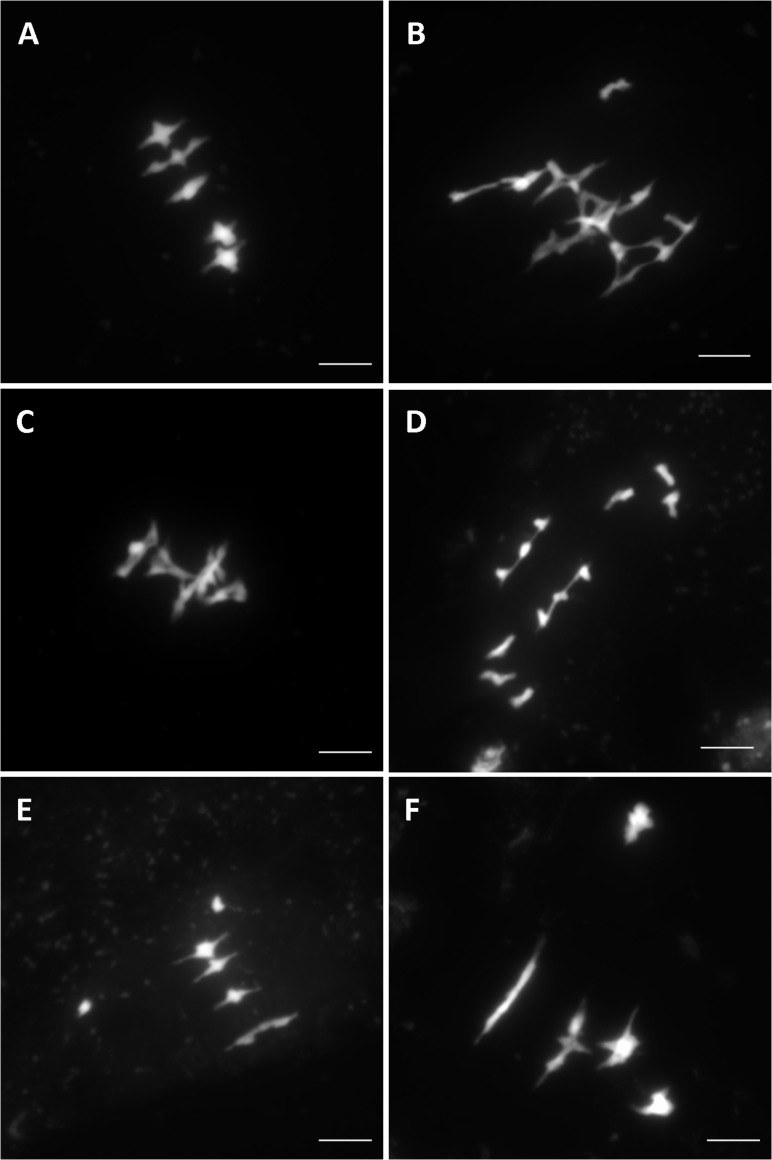
Different meiotic phenotypes observed in the HEM lines at metaphase I showing: **(A)** Wild type meiosis **(B)** Chromosome fragmentation, **(C)** Bivalent shape defects, **(D,E)** Different levels of univalent chromosomes (ranging from a mean of 6 to 0.1 per cell) and **(F)** Bivalent alignment defects.

Overall, after screening 80% of the HEM lines the rate of meiotic mutant was high, with 9.4% of the lines showing different types of meiotic phenotypes in the DH and SSD series 10 (43 mutants with a robust meiotic phenotype among 199 DH lines + 261 series 10 SSD lines). The phenotypes described in the 43 HEM lines identified cover a variety of meiotic defects at metaphase I, compared to wild type (**Figure 3A**): (i) Different levels of fragmentation (suggesting a failure to complete the recombination process and leading in some cases to reduced fertility; observed in 10 lines; **Figure 3B**), (ii) bivalent shape defects (observed in five lines; **Figure 3C**), (iii) the presence of univalent chromosomes at different frequencies suggesting a lack of crossovers (ranging from 0.1 to 6 pair of univalent chromosomes per cell; observed in 26 lines; **Figures 3D,E**), and (iv) bivalent alignment abnormalities (observed in 2 lines; **Figure 3F**).

### Identification of Candidate Causal Mutations

Of the 41 lines sequenced with meiotic defects, 18 had an obvious candidate mutation (**Table 3**). We considered a candidate mutation to be causal when the mutation was predicted to strongly affect a protein (i.e., stop codon, frame shift or a splice site change) with a described role in meiosis. Additionally, the observed phenotype had to be consistent with the previously described phenotype. An additional missense mutation in *ATM* was shown to be causal by genetic mapping (**Table 3**). In all these lines, the presence of the candidate mutation was confirmed by Sanger sequencing. In addition, Sanger sequencing of the candidate gene *SGO1* in two lines that showed premature loss of sister chromatid cohesion identified a stop and a splice site mutation (lines HDGem3 and HD479; **Table 3**).

**Table 3:** List of the genes identified as candidate causal mutations in the HEM lines.

Gene	Function	Position in bp (TAIR 10)	Mutant line	Change	Mutation effect	Phenotype	References
ATM (AT3G48190) Chr3	DSBs repair	17807938	ES1M5S10236	G > A	Premature stop	Fragmentation	Garcia et al., 2000, 2003
		17827101	ES1M5S10017	G > A	Splice change	Fragmentation	
		17824467	ES1M5S10070	G > A	Missense	Fragmentation	
		17812658	HD776	G > A	Splice change	Fragmentation	
		17823207	HD790	G > A	Splice change	Fragmentation	
MLH3 (AT4G35520) Chr4	class I COs	16868001	ES1M5S10052	C > T	Premature stop	Univalent chromosomes	Jackson et al., 2006
		16868745	HD662	C > T	Premature stop	Univalent chromosomes	
Mer3 (AT3G27730) Chr3	class I COs	10277172	ES1M5S10068	C > T	Splice change	Univalent chromosomes	Chen et al., 2005; Mercier et al., 2005
		10278220	HD577	C > T	Splice change	Univalent chromosomes	
Hei10 (AT1G53490) Chr1	Class I COs	19964116	HD768	G > A	Start gained	Univalent chromosomes	Chelysheva et al., 2012
FLIP (AT1G04650) Chr1	Pro and anti- CO	1298121	ES1M5S10083	C > T	Splice change	Univalent chromosomes	Fernandes et al., 2018
FANCL (AT5G65740) Chr5	Class II COs	26302687	ES1M5S10195	G > A	Premature stop	Univalent chromosomes	Girard et al., 2014
PRD2 (AT5G57880) Chr5	DSBs formation	23446256	ES1M5S10108	G > A	Premature stop	All univalent chromosomes	De Muyt et al., 2009
Rec8 (AT5G05490) Chr5	Cohesin subunit	1625685	ES1M5S10121	G > A	Splice change	Fragmentation	Chelysheva et al., 2005
ASY4 (AT2G33793) Chr2	CO formation and synapsis	14297325	HD721	G > A	Splice change	Univalent chromosomes	Chambon et al., 2018
PSS1 (AT3G63480) Chr3	Synapsis and CO regulation	23443192	HD783	C > T	Splice change	Univalent chromosomes	Duroc et al., 2014
MLH1 (AT4G09140) Chr4	COs class I	5820399	HD803	C > T	Splice change	Univalent chromosomes	Dion et al., 2007
	COs class I	5820399	HD853	C > T	Splice change	Univalent chromosomes	
SGO1 (AT3G10440) Chr3	Chromosome cohesion	3246274	HDsach17	G > A	Splice change	Univalent chromosomes	Cromer et al., 2013
		3246364	HDGem3	G > A	Splice change	Univalent chromosomes	
		3248339	HD479	C > T	Premature stop	Univalent chromosomes	

*The table includes information on the name of the gene, meiotic function, position of the mutation, the nucleotide change resulting from the mutation, the effect of the mutation, the phenotype showed by the mutant and the literature describing these mutants.*

The genes described in the 21 lines are involved in a wide range of meiotic mechanisms: *ATM* (found in five different mutant lines) plays a role in DSBs repair (Garcia et al., 2000, 2003); *MLH1*, *MLH3*, *MER3* (each identified in two different mutant lines), and *HEI10* are involved in class I crossover formation (Mercier et al., 2005; Jackson et al., 2006; Dion et al., 2007; Chelysheva et al., 2012), *FLIP* is both an anti- and pro-crossover factor (Fernandes et al., 2018); *FANCL* promotes crossover formation (Girard et al., 2014); *PRD2* is required for DSBs formation (De Muyt et al., 2009); *REC8* and *SGO1* (found in 3 different mutant lines) are both involved in sister chromatid cohesion (Chelysheva et al., 2004; Cromer et al., 2013); *ASY4* is involved in crossover and synaptonemal complex formation (Chambon et al., 2018); and *PSS1* plays a role in chromosome synapsis and crossover distribution (Duroc et al., 2014; **Table 3**). Interestingly, most of these mutations have only a mild impact on fertility (e.g., *MLH1, MLH3, ASY4, FANCL*, or *PSS1*).

In addition, among the 43 lines identified with meiotic phenotypes, in 22 there was no obvious candidate mutation, according to the criteria described above. These lines displayed different meiotic phenotypes and further work is needed to identify their causal mutation.

## Discussion

We have described the HEM collections, two libraries of almost fully homozygous *Arabidopsis* EMS mutants. These mutants show a high mutation rate per line, 897 mutations per line on average, of which most are fixed. Thus, due to the fixed nature of the mutations, these libraries can be used to repeatedly screen for a specific phenotype and therefore, to analyze either quantitative or qualitative traits.

Of all the mutations produced, we estimate that the HEM lines contain, >170,000 mutations (195 per line, on average) with an effect in the protein sequence. Among these >12,000 (14 per line, on average) are mutations that likely knock out the protein’s function (new stop codons, splice site changes and frameshifts).

In this study, the HEM lines were used in a forward screen targeting subtle meiotic phenotypes as a new approach to identify novel meiotic mutants. Nine Percent of the mutant lines screened in the DH and SSD series 10 collections showed defects in meiosis (43 lines), which is a direct evidence of the efficiency of mutagenesis in the HEM lines.

Within these mutants, there are 12 clear candidate genes (*ATM, MLH3, MLH1, MER3, HEI10, SGO1, ASY4, FLIP, FANCL, PRD2, REC8*, and *PSS1*) involved in different meiotic mechanisms. Interestingly, six of these identified genes (*MLH3, MLH1, SGO1 PSS1, FANCL*, and *ASY4*) have been found here for the first time in a forward genetic screen. These mutants have only moderate defects in chromosome distribution at meiosis, leading to a subtle reduction in fertility, which is under the threshold of detection by visual examination of fruit length.

In addition, the finding that in 22 mutant lines there is no obvious causal mutation among the previously described meiotic genes, suggests that these lines may be mutated in novel meiotic genes that will require genetic mapping to be identified. Thus, these results are a proof of concept and support the usefulness of the HEM lines to decipher various biological processes. The two collections are available at the Versailles *Arabidopsis* stock center.

## Materials and Methods

### EMS Mutagenesis and Plant Growth

To generate the single seed descent (SSD) collection, we applied ethyl methanesulfonate (EMS) to wild type *A. thaliana* accession Col-0 as described in (Portemer et al., 2015). Seeds were incubated for 17 h at room temperature with gentle agitation in 5 mL of 0.3% (v/v) EMS. Neutralization was performed by adding 5 mL of sodium thiosulfate 1 M for 5 min. Three milliliter of water was added to make the seeds sink. The supernatant was removed and the seeds were washed three times for 20 min with 15 mL of water. Mutagenized seeds were grown and carried through to the fourth generation using only one seed each time. The M4 seeds were used to screen for meiotic defects.

To generate the DH collection, mutagenesis was performed as in the SSD in Col-0 plants with an existing T-DNA insertion in *GLABRA1* (*GL1*). Mutagenized seeds were grown and then crossed as male to the tailswap line (TS) to obtain haploid plants that could be identified due to their lack of trichomes. Diploids were obtained by self-fertilization. M4 seeds were multiplied to obtain the final mutant population of the collection. All plants were cultivated in greenhouses with a 16 h/day and 8 h/night photoperiod, at 20°C and 70% humidity.

### Plant Phenotyping

Alexander staining for pollen viability was performed as described in Alexander (1969). Meiotic chromosomal spreads were prepared and stained with DAPI as described in Ross et al. (1996). Observations were made using a Zeiss Axio Observer epifluorescence microscope and photographs were taken using an AxioCam MRm (Zeiss) camera driven by ZEN 2 Software (Carl Zeiss Microscopy, GmbH). Plots and statistical analysis were made using the GraphPad software Prism6^1^.

### Whole Genome Sequencing and Mutation Analysis

Genome sequencing was performed with Illumina Hiseq3000 HWIJ00115 with > 8X coverage. The resulting fastq files were analyzed using the Mutdetect pipeline (version 0.0.6-e3ef10e) 1http://www.graphpad.com

(Girard et al., 2015) using TAIR10 COL-0 genome as the reference genome. The FileMatch package was used to eliminate false positives by comparing each muther two mutant lines as controls. Mutations were considered after quality filtering (>80) and the presence of 0 or only one read with wild type allele was considered to indicate a homozygous mutations. Additionally, to differentiate real mutations from false positives, we compared the total number of reads with the coverage, discarding mutations that showed unmapped reads as a proxy for repetitive regions. The sequencing raw data of fully characterized lines is available in the sequence read archive at NCBI SRA accession: SRP156100) and we encourage future users of the collection to do the same.

## Author Contributions

LC-P, VP, MG, and RM contributed to the conception and design of the study. LC-P, VS, AC, AH, AG, VP, DV, and LC performed the experiments. LC and RM analyzed the sequencing data. LC-P wrote the first draft of the manuscript.

## Conflict of Interest Statement

The authors declare that the research was conducted in the absence of any commercial or financial relationships that could be construed as a potential conflict of interest.

## Footnotes

1 http://www.graphpad.com
